# HIGH-RISK ELDERLY SCREENING IN MEDICAL INTENSIVE CARE UNIT

**DOI:** 10.1093/geroni/igac059.2029

**Published:** 2022-12-20

**Authors:** Gowrishankar Gnanasekaran, Narendrakumar Alappan

**Affiliations:** Cleveland Clinic Foundation, Cleveland, Ohio, United States; Fairview Hospital, Cleveland Clinic, Cleveland, Ohio, United States

## Abstract

Geriatrics care in medical intensive care units (MICU) establishes a unique opportunity in early screening of High Risk Elderly (HRE) patients admitted for critical care. Many MICUs do not have a standard protocol to screen for HRE patients as part of their daily huddle. Our program is a quality improvement initiative to improve early identification of HRE patients in the MICU. HRE patients were identified based on nursing specific screening triggers at one of the Regional Hospitals of a large teaching hospital in Northeast Ohio.The program was designed as a part of geriatrics care expansion at regional hospital sites. Identified patients were discussed in daily huddles to determine unique geriatric needs in caring for these patients. A geriatrics co-management team was engaged in comprehensive geriatric assessments and care transition when it was needed. Geriatrics care in MICU demonstrates a unique opportunity in early identification of HRE patients. This helps to support a patient -centered approach in caring for critically ill elderly patients. The program would lay foundations in early screening for risk factors and optimizing elderly care in MICU.

